# Wnt5a / planar cell polarity signaling pathway in urothelial carcinoma, a potential prognostic biomarker

**DOI:** 10.18632/oncotarget.15877

**Published:** 2017-03-03

**Authors:** Mark Saling, Jordan K Duckett, Ian Ackers, Karen Coschigano, Scott Jenkinson, Ramiro Malgor

**Affiliations:** ^1^ Department of Biomedical Sciences, Heritage College of Osteopathic Medicine, Ohio University, Athens, Ohio, USA; ^2^ Interdisciplinary Program in Molecular and Cellular Biology, Ohio University, Athens, Ohio, USA; ^3^ The Diabetes Institute of Ohio University, Athens, Ohio, USA; ^4^ University Medical Associates, Inc., Pathology, Athens, Ohio, USA

**Keywords:** urothelial carcinoma, bladder, Wnt5a, epithelial mesenchymal transition, biomarker

## Abstract

Bladder cancer is the fourth most common cancer in men and the most common malignancy of the urinary tract. Bladder cancers detected at an early stage have a very high five-year survival rate, but when detected after local metastasis the rate is only about 50%. Our group recently reported a positive correlation between the expression of Wnt5a, a member of the Wnt proteins family, and histopathological grade and stage of urothelial carcinoma (UC). The objective of this study was to analyze UC cases reported in Athens, Ohio and investigate the major components of Wnt5a / planar cell polarity (PCP) signaling pathway in UC human tissue samples and UC cell lines.

Formalin fixed and paraffin embedded transurethral resection tissues were immunostained for Wnt5a, Ror-2, CTHRC1 and E-cadherin. In addition, *in vitro* studies using UC cell lines were investigated for Wnt5a/PCP signaling and epithelial mesenchymal transition (EMT) gene expression. The IHC results showed a correlation between the expression of Wnt5a, Ror2 and CTHRC1 with high histological grade of the tumor, while E-cadherin showed an opposite trend of expression. Real time RT-PCR results showed that RNA expression of the Wnt5a/ PCP pathway genes vary in low and high grade UC cell lines and that the high grade cell lines exhibited signs of EMT.

These findings support that Wnt5a-Ror2 signaling plays a role in UC, support the potential use of Wnt5a as a prognostic marker and provide evidence that Wnt5a signaling may be used as an effective molecular target for novel therapeutic tools.

## INTRODUCTION

Bladder cancer is the fourth most common cancer among men. About 74,000 new cases are diagnosed, and result in 16,000 deaths each year in the United States [1]. Many bladder cancers at first diagnosis are found still confined to the superficial layer of the bladder wall, but 35% of the diagnoses have invaded into deeper layers including muscle. Approximately 95% of bladder cancers arise from the urothelium, causing urothelial carcinoma (UC) [2, 3].

The pathological grade is essential criteria for determining prognosis and patient therapeutic management [3, 4]. Urothelial carcinoma is diagnosed and treated via transurethral resection (TUR). The World Health Organization (WHO) devised a 2 tier system in 2004, distinguishing low grade and high grade UC tumors. Low grade tumors maintain recognizable architecture with minimal changes in polarity, nuclear size, shape, and chromatin texture, while high grade tumors have disorganized architecture and frequent mitotic figures as well as increased pleomorphism and clumped chromatin [2]. The heterogeneous UC group includes tumors with different biological behaviors, and recurrence after the initial treatment is a common feature [3].

Multiple biomarkers are currently being investigated in the use for diagnosis and prognosis of bladder cancer, including Wnt5a [5–7]. Wnt5a belongs to the Wnt family of proteins, which is a group of evolutionarily conserved glycoproteins shown to play a key role in embryonic development, regulating cell proliferation, fate, and motility [8, 9]. A peculiarity of the Wnt protein family is the large number of ligands and receptors involved, leading to the activation of multiple downstream pathways [8]. The pathways can be classified into two categories, canonical or transforming Wnts and non-canonical or non-transforming Wnts. Aberrant activation of Wnt signaling pathways have been involved in a large variety of diseases [10, 11]. Activation or inhibition of Wnt5a signaling, a member of the noncanonical Wnt signaling pathways has been described as an important event in pathogenesis of cancer as tumor suppressor or tumor promoter, in a variety of malignancies [12, 13]. Moreover, it has been shown that Wnt5a can work through canonical/β-catenin dependent or noncanonical/β-catenin independent signaling pathways [10, 14].

Wnt5a, can activate numerous pathways based on the use of a large variety of receptors and co-receptors, including the Frizzled receptor (Fzd) family, tyrosine kinase-like orphan receptor 2 (Ror2), and collagen triple helix repeat-containing protein 1 (CTHRC1) [15, 16], among others. Ror2, a member of the Ror family of receptor tyrosine kinases, is involved in Wnt/planar cell polarity (PCP) and has been shown to play a role in the epithelial-mesenchymal transition [17, 18]. CTHRC1 selectively activates the Wnt/PCP pathway through activation of RhoA and Rac, and it enhances the formation of the Wnt-Fzd-Ror2 complex [16]. It is thought that CTHRC1 stabilizes the Wnt ligand/Frizzled receptor complex during Wnt/PCP pathway activation [16]. CTHRC1 has been reported to be highly expressed in pancreatic cancer and hepatocellular carcinoma [19], but neither the role of Ror2 or CTHRC1 has been studied in UC.

Previously, we reported a positive correlation between Wnt5a expression and histopathological grade of UC in human samples [6]. This finding led us to speculate that Wnt5a could play a role in UC pathogenesis and could be a potential biomarker to explore for this type of cancer. The aim of this current study was to investigate Wnt5a/Ror2/PCP signaling as a potential pathway functioning in the pathogenesis of aggressive UC cases. In this study we found that Ror2 expression shows a similar pattern as Wnt5a in the tissue samples. More important was the finding that the correlation between Ror2 and pathological grade is even stronger than the correlation between Wnt5a and pathological grade. Additionally, we found that CTHRC1 is highly expressed in the tumors with high Ror2 expression. *In vitro* studies using three UC cell lines, RT4, J82 and T24, the first isolated from a low grade UC and the last two from high grade UC tumors, showed a similar trend of RNA expression for Wnt5a and Ror2. We conclude that the Wnt5a/PCP pathway may be playing an important role in aggressive cases of UC, providing potential applications for development of new diagnostic and treatment strategies for UC.

## RESULTS

### Epidemiologic study

**Figure 1:**
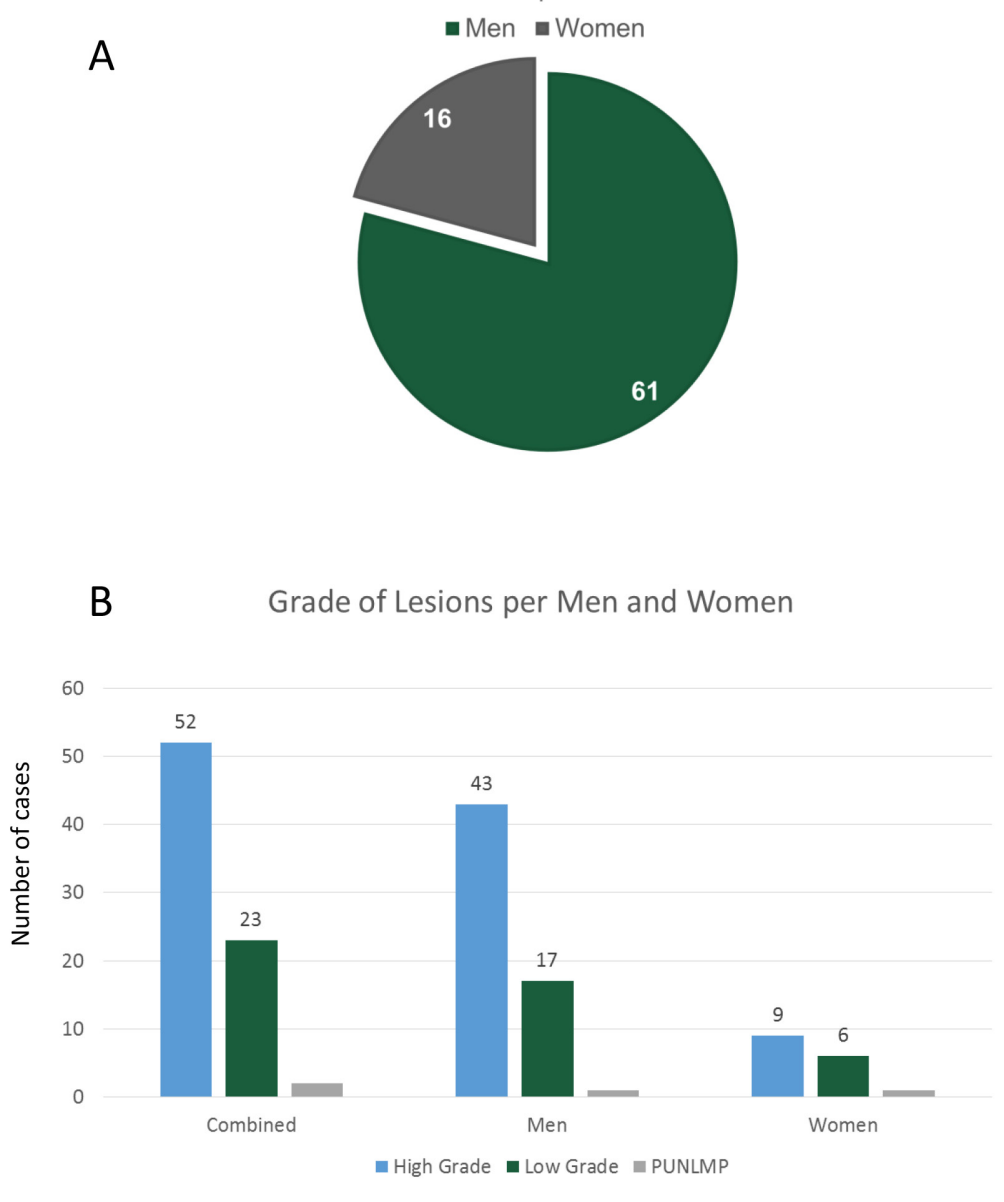
**(A)** Gender distribution. Of the 77 cases collected 61 (79.2%), were men and 16 (20.8%), were women. **(B)** Grade analysis: Comparison of high grade to low grade tumors for male and females. In this population, the likelihood to have high grade or low grade tumors were 2.5 and 1.5 for men and women respectively.

For the period 2004-2014, 77 cases were diagnosed as UC through the UMA Pathology lab and reviewed in this study. A total of 75 cases where confirmed as UC with 52 (69.3%), being high grade (HG) lesions and 23 (30.7%), being low grade (LG) lesions. Two other cases (2.6%) were diagnosed as papillary urothelial neoplasm of low malignant potential (PUNLMP). The average patient age for all cases was 71.9 years. Figure 1A shows the male-dominant, 61 of 77 (79.2%), gender distribution of the 77 cases collected. In the group studied with the diagnosis of UC the likelihood for men and women to have HG versus LG tumors were 2.5 and 1.5 times, respectively (Figure 1B).

### Histopathological analysis of human UC samples

Histological grade was determined for 15 samples of human UC included in the study. Based on the pathological reports 7 samples were low grade and 8 were high grade urothelial carcinomas. Of the 15 samples, 5 (4 high grade, 1 low grade) showed muscular invasion and thus were at least stage T2. The other 10 cases only invaded the lamina propria. Based on microscopic appearance 3 of the invasive tumors showed a solid pattern, while the rest (n=12) showed papillary aspect.

### Wnt5a and Ror2 protein expression correlated with increasing histological grade

**Figure 2:**
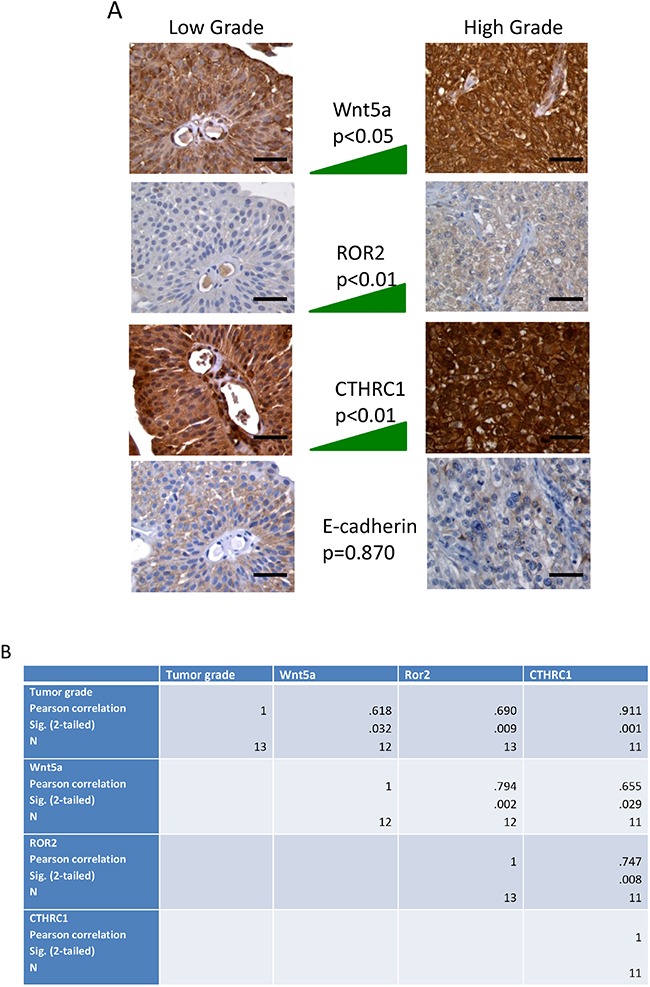
**(A)** Immunohistochemical analysis of human specimens of urothelial carcinoma of the bladder for the expression of Wnt5a, ROR2, CTHRC1 and E-cadherin. Left column, tissue sections from a representative case of low grade urothelial carcinoma; the right column, tissue sections from a representative case of high grade urothelial carcinoma. The middle column represents the trend of expression for each protein in all 15 samples. The expression of Wnt5a, ROR2, and CTHRC1 increases in high grade tumors while E-cadherin shows an opposite trend. Bar=50 μm. **(B)** Statistical analysis was performed to investigate the correlation between tumor histological grade and immunostaining for Wnt5a, Ror2, CTHRC1, and E-cadherin. Statistical significance was tested at an alpha of 0.05. The software PASW Statistics 18 was used for data analysis (Pearson Education, New York City, NY).

Immunohistochemical analysis revealed a statis-tically significant correlation between Wnt5a protein expression and tumor grade (Sig. 0.032) (Figure 2A and 2B). This result supported our previous study [6]. In addition, we found a higher correlation between Ror2 expression in the tumor and tumor grade and between Ror2 and Wnt5a protein expression in tumor tissue, (Sig. 0.009 and 0.002), respectively (Figure 2B). Correlation between Wnt5a and CTHRC1 was significant (0.029). Correlation between Ror2 and CTHRC1 expression was also highly significant (0.008). The expression of Wnt5a, ROR2, and CTHRC1 increased with the histological grade while E-cadherin showed an opposite trend (Figure 2A).

### Varying combinations of expression of Wnt5a, Ror2 and CTHRC1 in three UC cell lines

**Figure 3:**
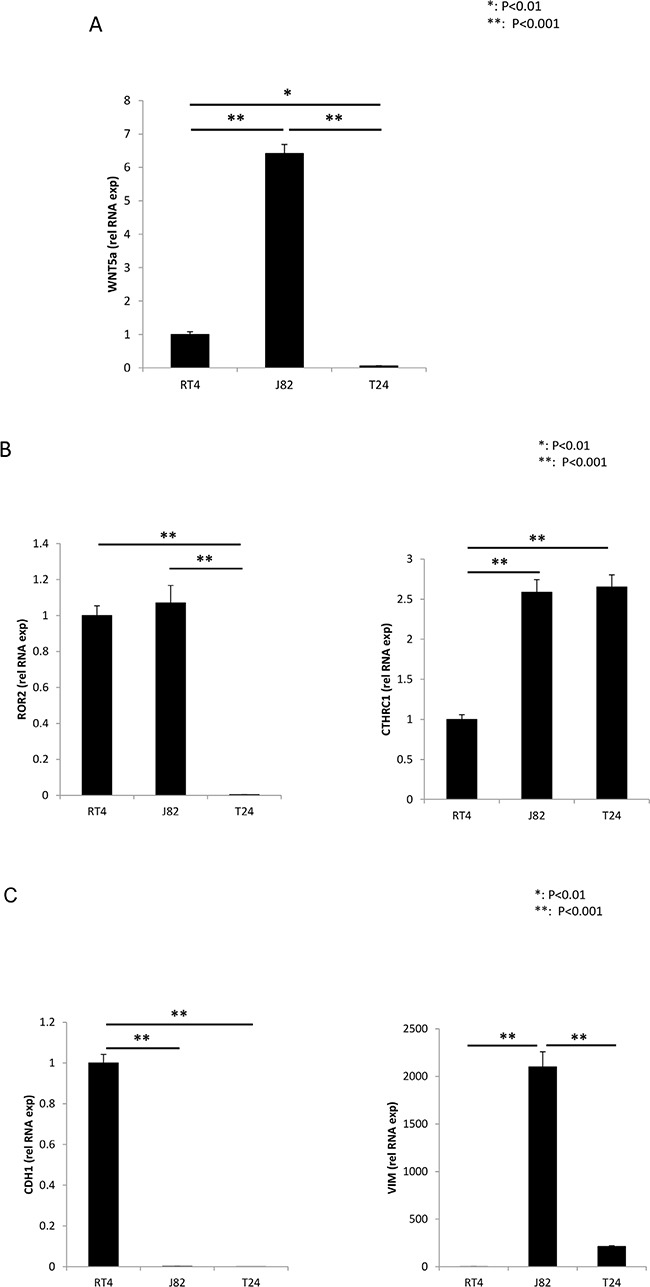
Real Time RT-PCR, analysis of RNA expression Wnt5a **(A)**, Ror2 and CTHRC1 **(B)**, E-cadherin and vimentin **(C)**, in RT4, J82 and T24 urothelial carcinoma cell lines.

**Figure 4:**
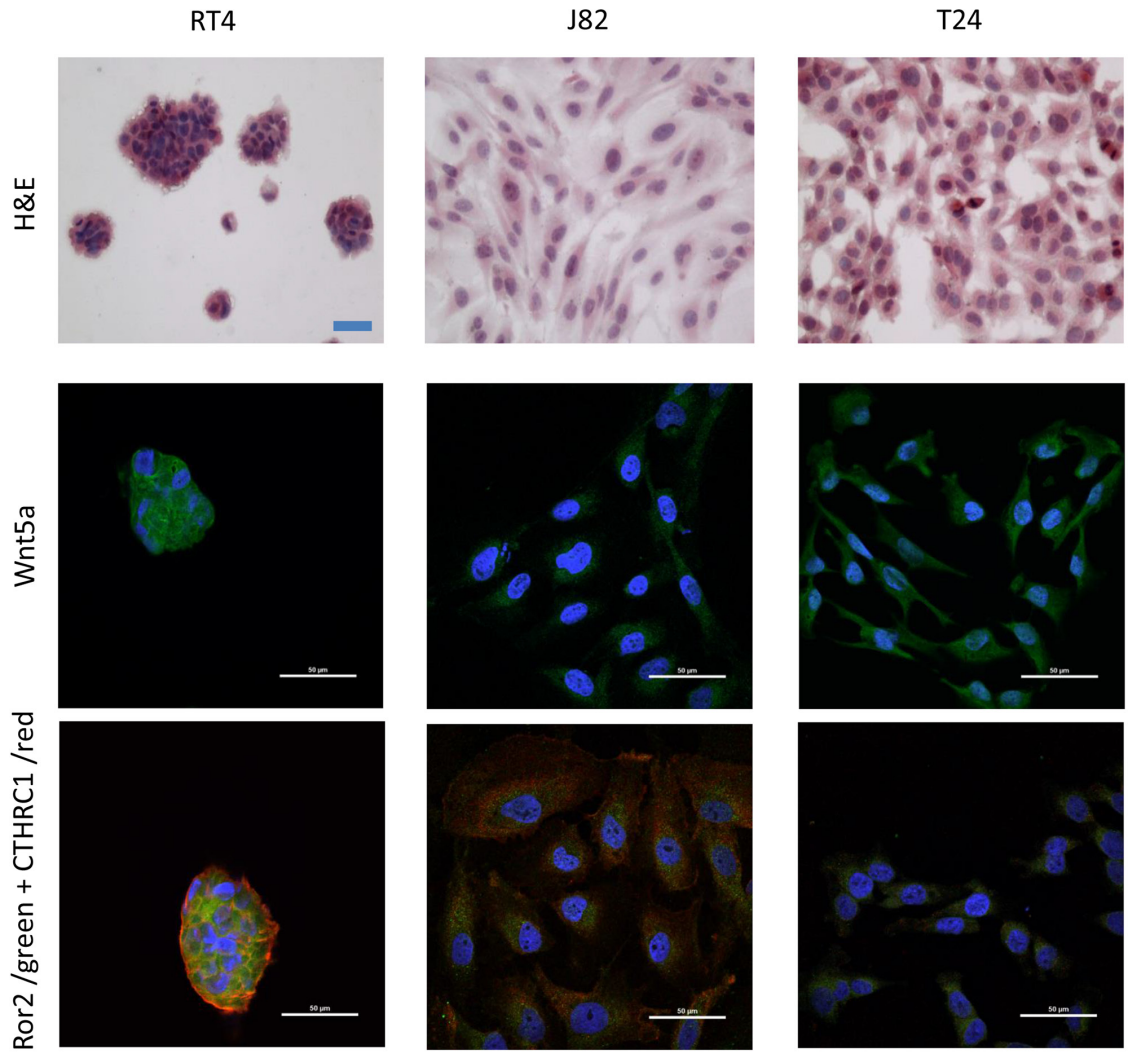
Immunofluorescence of RT4, J82 and T24 cell lines for the expression of Wnt5a, ROR2, and CTHRC1 Top row: H&E stain shows morphological differences between each cell line. Middle row: confocal microscopy images show the expression of Wnt5a (green) for each cell line. Bottom row: confocal microscopy image of the merge expression of Ror2 (green) and CTHRC1 (red) for each cell line. Although the co-expression and colocalization of Ror2 and CTHRC1 is present in all cell lines, it is clearest for RT4 and J82 cell lines.

Real-time RT-PCR analyses of RNA isolated from RT4, J82 and T24 cells revealed dramatically different levels of expression of Wnt5a between the three cell types (Figure 3A). In comparison to expression in the low grade RT4 cell line, Wnt5a RNA expression was dramatically increased in the high grade J82 cell line and severely decreased in the high grade T24 cell line. Interestingly, Ror2 expression paralleled Wnt5a expression in the high grade J82 and T24 cell lines, but was also high in the low grade RT4 cell line (Figure 3B). CTHRC1 expression was high in both high grade cell lines, J82 ant T24, in comparison to the low grade RT4 cell line (Figure 3B). Immunofluorescence analysis of protein expression within the cell lines roughly paralleled RNA expression for Wnt5a and CTHRC1. In contrast, Ror2 protein expression appeared to be low in the high grade J82 cell line, in contrast to the high RNA expression. The confocal images show co-expression and co-localization of Ror2 and CTHRC1 in RT4 and J82, being less evident for T24 cell line (Figure 4).

### RNA expression of CDH1 and vimentin suggest an epithelial-mesenchymal transition for the high grade UC cell lines

As expected for an epithelial cell, RNA expression of CDH1 was easily detected, but vimentin levels were extremely low in the low grade RT4 cell line (Figure 3C). In contrast, RNA expression of vimentin was greatly increased in the high grade J82 cell line and detectable, but not significantly increased in the high grade T24 cell line in comparison to the low grade RT4 cell line; CDH1 expression was extremely low in both high grade cell lines (Figure 3C).

## DISCUSSION

The Ohio Cancer Incidence Surveillance System (OCISS) reported 4,669 new cases of bladder cancer for the period 1999-2007 and 2,806 new cases for the period 2006-2010 [20]. At the national level, according to the American Cancer Society, there was an expected 74,690 new cases of bladder cancer for the United States and for Ohio an estimated 3,110 new cases for 2014. In our analysis of 77 UC cases reviewed for a 10 year period (2004-2014) in Southeast Ohio, the male to female ratio was 3.8:1, and the mean age was 72 years, in agreement with previous studies [4]. Although UC is not a cancer with high mortality rate such as breast, colon or lung cancer, it has many biological and clinical features that make it interesting to study. First, bladder cancer detected at an early stage has a five year survival rate of around 92%, but when detected after local metastasis the rate is only about 50% [21]. For this reason, early detection is an important area of investigation. Second, UC has a high percentage of intravesical recurrence (30-80%) after TUR treatment, even for non-muscle-invasive UC cases [21].

The search for new biomarkers for accurate prediction of UC outcome is a current challenge [5, 7]. In this context, our lab is focused on identification of new biomarkers for UC. Previously, we reported a positive correlation between the expression of Wnt5a and histopathological grade and stage of UC [6]. Our results suggest a potential value of Wnt5a signaling as a prognostic marker for UC.

In this study, we reported the expression of Wnt5a, Ror2 and an additional co-factor, CTHRC1, in the UC samples and urothelial cancer cell lines. The expression of CTHRC1 and the positive correlation between Wnt5a/Ror2 and pathological grade suggests that the Wnt5a/PCP signaling pathway could play a role in the aggressiveness of this cancer.

Expression of Wnt5a has been reported in several types of cancers, but the role of Wnt5a in the pathogenic mechanisms of cancer is still unclear, with opposing roles as tumor promoter and tumor suppressor being described [22, 23]. Recently, Wnt5a/Ror2 signaling has been implicated as an important pathway in epithelial-mesenchymal transition and promotion of invasion and metastasis in pancreatic cancer [24]. CTHRC1 is known as a regulator of collagen expression and cell migration [23, 25]. The significance of CTHRC1 in the pathogenesis of cancer is still unclear, but its overexpression has been reported associated with poor prognosis in solid tumors, gastric and breast cancer [26–28]. Moreover, the inhibition of CTHRC1 using siRNA has been described as potential therapeutic strategy for cancer progression [12].

Interestingly, our results show a correlation between the expression of CTHRC1 and tumor grade in TUR samples, and the most aggressive of the three cell lines included in this study, J82, also expressed high transcription level of CTHRC1 gene. By immunofluorescence, presence of the Wnt5a protein in the three UC cell lines is clear. Even more interesting, confocal microscopy demonstrates the co-localization of Ror2 and CTHRC1, both receptors described in the non-canonical cell polarity pathway, most obvious in the J82 cell line. These results provide evidence for the potential activation of the Wnt5a/PCP pathway during UC pathogenesis. Taking into consideration all the above, our results suggest the potential role of Wnt5a/PCP pathway through the Ror2 signaling pathway in the development, progression and metastasis of bladder urothelial carcinoma.

Recently, expression of EMT-associated markers has been reported as potential predictors of recurrence in cases of non-muscle-invasive UC after TUR [29]. Specifically, E-cadherin has been reported as a potential prognostic marker for UC [29, 30]. In line with these findings, our data shows that high grade UC cell line, J82 expressed high levels of Wnt5a and vimentin mRNA, but low levels of E-cadherin mRNA, suggesting a role of Wnt5a signaling in EMT in UC. Recently, Twist and Wnt signaling pathways have been associated with UC and metastatic breast cancer via activation of EMT genes transcription suggesting a potential therapeutic use by interference of these pathways [29, 31].

In conclusion, these data support our previous studies that suggest that the Wnt5a signaling pathway plays a pathological role in UC. A correlation between Wnt5a/Ror2 and pathological grade suggests that Wnt5a/Ror2 signaling pathway could play a role in the aggressiveness of this cancer, perhaps promoting the EMT and metastasis process. The results also support their potential use as molecular biomarkers and therapeutic target for UC. Further studies are needed to determine the underlying mechanism of Wnt5a/Ror2 action in the pathogenesis/progression of UC as well as their application as biomarkers for UC.

## MATERIALS AND METHODS

### Epidemiologic study of human urothelial carcinoma

Reports of patients with initial diagnosis of bladder tumor at the University Medical Associates Pathology Department (UMA Pathology lab, Athens, OH) during a 10 year period (2004-2014) were reviewed. This study was performed after obtaining approval by the Ohio University Institutional Review Board (IRB 07E112). All cases were re-evaluated to confirm the initial diagnosis and each case was recorded and categorized by age, and sex. The diagnosis, determination of histological grade and pathological stage were verified on hematoxylin and eosin (H&E) stained sections, based on the WHO/ISUP consensus classification system [2, 32]. Histological grade was determined by evaluating tissue architecture, polarity, and cohesiveness, as well as cytological features such as pleomorphism, chromasia, and mitosis as described previously [2]. Pathological stage was determined by the depth of invasion into the lamina propria and muscularis propria. Descriptive statistics focused on frequencies and proportions for variables in this group (n= 77) was performed.

### Protein expression in UC samples by immunostaining

Immunohistochemial staining of 15 formalin fixed paraffin embedded (FFPE), TUR samples, collected in 2013-2014, was performed. These 15 samples were included in the total of 77 cases reviewed for the epidemiologic study. The immunostaining was performed as previously described [6]. Briefly, for each case, two 4 μm sections were mounted onto a Superfrost glass slide. Sections were deparaffinized by xylene and rehydrated in decreasing concentrations of ethanol. After antigen retrieval using 10 mM citrate buffer, pH 6.0, at 90°C for 30 minutes, the endogenous peroxidase activity was blocked with 3% H_2_O_2_ in phosphate buffered saline (PBS). To one of each of the two sections the following antihuman primary antibodies were applied: mouse monoclonal antibody against Wnt5a (Abcam, Cambridge, MA) applied at 8.33 μg/ml; rabbit polyclonal antibody against Ror2 (Abnova, Taipei City, Taiwan) applied at 1/100 dilution, and mouse monoclonal antibody against CTHRC1 (Abnova, Taipei City, Taiwan) applied at 4.0 μg/ml. All primary antibodies were diluted in 1% bovine serum albumin (BSA) in PBS and incubated overnight at 4°C in a humidified chamber. For each primary antibody the corresponding isotype control was applied at a similar concentration to the consecutive section on each slide; normal mouse IgG (Santa Cruz Biotechnology, Santa Cruz, CA) was applied as isotype control for Wnt5a and CTHRC1 and normal rabbit IgG (Santa Cruz Biotechnology, Santa Cruz, CA) as isotype control for Ror2.

The next day, after three washes with PBS, the slides were incubated with goat anti-mouse IgG (Abcam, Cambridge, MA) or goat anti-rabbit IgG (Abcam, Cambridge, MA) HRP labeled for 1 hour at room temperature. Following four washes with PBS, diaminobenzidine (DAB) chromogen (ThermoScientific, Waltham, MA) was applied for 3 minutes, and then the sections counterstained with hematoxylin, dehydrated and mounted with a coverslip.

The immunostaining for each antibody was scored in a semi-quantitative manner according to Allred *et al*. [33], briefly, based on intensity (intensity score) and extent (proportion score) of stained tumor cells viewed at 400x in 10 fields per section by two independent observers according to the following criteria: intensity; 0, weak or no staining, up to 3+, most intensive staining; proportion: 0, less than 25% of the tumor cells were stained; 1+, 25% to 50% of the tumor cells were stained; 2+, moderate, 50% to 75% of the tumor cells were stained; 3+, more than 75% of the tumor cells were stained. Cases with discrepant scores were re-evaluated jointly until agreement between observers was reached.

### *In vitro* studies

In order to confirm our results on TUR samples collected from patients, we worked with three immortalized commercially available UC cell lines well known and characterized [34–36]: RT4 (ATCC ®HTB-2™), isolated form transitional cell papilloma and classified as differentiated low grade UC; J82 (ATCC HTB-1™), derived from UC high histological grade that infiltrated into deep muscle (Stage T3); and T24 (ATCC HTB-4™), derived from UC high histological grade (Table 1).

**Table 1:** Characterization of human urothelial carcinoma cell lines

Cell line	Origin of tumor cells*	Clinical stage of disease	Histological grade*	Tumorigenic*	Wnt5aRNAexpression#	Ror2RNAexpression#	CTHRC1RNAexpression#	E-cadherinRNAexpression#	VimentinRNAexpression#
RT4	recurrent in bladder	T2	Low grade(G1)	Yes	+	+	+	+	+
J82	bladder primary	T3	High grade(G3)	Yes	highly increased	increased	increased	decreased	highly increased
T24	recurrent in bladder	NR	High grade(G3)	No	decreased	highly decreased	increased	decreased	increased

*Cancer Res 46, 3630-36, July 1986 [19]

#relative to RT4

### Real-time RT-PCR analysis

Total RNA was isolated from replicate cultures of J82, T24 and RT-4 cells grown to near confluency in six well plates (N= 6 for each) using TRIzol Reagent (Life Technologies Corporation, Carlsbad, CA). RNA samples were treated with DNase I to remove contaminating genomic DNA and repurified using the RNA Clean & Concentrator-5 kit supplied with DNase I according to the manufacturer's instructions (Zymo Research Corp, Irvine, CA). Purified RNA was quantified using a Nanodrop 1000 spectrophotomenter (Thermo Scientific, Wilmington, DE). cDNA was synthesized from 1 μg of RNA per 20 μl cDNA reaction using the iScript cDNA Synthesis Kit (Bio-Rad, Hercules, CA). cDNA samples were assayed for relative target concentration via real-time RT PCR analysis using gene-specific primers (sequences in Table 2 were obtained from the PrimerBank database [37] or designed using the Primer-BLAST program [38]; primers were synthesized by integrated DNA Technologies, Inc., Coralville, IA) and the iTaq Universal SYBR Green Supermix with ROX and fluorescein reference dyes (Bio-Rad). Reactions were performed in duplicate amplifying 0.8 μl of undiluted template cDNA in a total reaction volume of 20 μl per sample using an annealing temperature of 55°C. Cycle threshold (Ct) values were converted to linear quantity values according to the delta Ct method [39]. Quantity values were then normalized for each sample and gene of interest by dividing each value by the geometric mean of the corresponding quantity values for the two most stable housekeeping genes, GAPDH and UBC out of five assessed (also HPRT1, SDHA, and B2M; Table 2) using a model-based variance estimation approach [39]. Normalized values for each gene of interest were finally compared between groups, relative to RT4.

**Table 2:** Primer sequences used for real-time RT-PCR analyses

Gene	GenBank accession number	Primer sequences (5′- 3′)	Primer design*
Glyceraldehyde-3-phosphate dehydrogenase (GAPDH)	NM_001256799	(+) CTGGGCTACACTGAGCACC (-) AAGTGGTCGTTGAGGGCAATG	PrimerBank ID 378404907c3
Beta-2-microglobulin (B2M)	NM_004048	(+) GAGGCTATCCAGCGTACTCCA (-) CGGCAGGCATACTCATCTTTT	PrimerBank ID 37704380c1
Hypoxanthine phosphoribosyltransferase 1 (HPRT1)	NM_000194	(+) CCTGGCGTCGTGATTAGTGAT (-) AGACGTTCAGTCCTGTCCATAA	PrimerBank ID 164518913c1
Succinate dehydrogenase complex, subunit A, flavoprotein (SDHA)	NM_004168	(+) CAAACAGGAACCCGAGGTTTT (-) CAGCTTGGTAACACATGCTGTAT	PrimerBank ID 156416002c1
Ubiquitin C (UBC)	NM_021009	(+) CTGGAAGATGGTCGTACCCTG (-) GGTCTTGCCAGTGAGTGTCT	PrimerBank ID 305632811c1
Wingless-type MMTV integration site family, member 5A (WNT5A)	NM_003392	(+) TCGACTATGGCTACCGCTTTG (-) CACTCTCGTAGGAGCCCTTG	PrimerBank ID 371506361c3
Receptor tyrosine kinase-like orphan receptor 2 (ROR2)	NM_004560	(+) TCCGAACGACCCTTTAGGAC (-) TTTAGCCACCGCACGTTAGG	PrimerBank ID 317008621c1
Collagen triple helix repeat containing 1 (CTHRC1)	NM_001256099	(+) TGTTCAGTGGCTCACTTCGG (-) TCCAGCACCAATTCCTTCACA	PrimerBLAST
Cadherin 1, type 1, E-cadherin (epithelial) (CDH1)	NM_004360	(+) AAAGGCCCATTTCCTAAAAACCT (-) TGCGTTCTCTATCCAGAGGCT	PrimerBank ID 169790842c3
Vimentin (VIM)	NM_003380	(+) AGTCCACTGAGTACCGGAGAC (-) CATTTCACGCATCTGGCGTTC	PrimerBank ID 240849334c2

*PrimerBank (Spandidos et al., 2010) [37]; PrimerBLAST (Ye et al., 2012) [38]

### Immunofluoroscence

Three urothelial carcinoma cell lines HTB-4™ (T24), HTB-1™ (J82) and HTB-2™ (RT4) (ATCC, Manassas, VA), were cultured according to ATCC recommendations or as described previously by Malgor *et al*. [6]. The cells were grown in chamber slides for 24 hours and after two washes with cold PBS, the cells were fixed in 4% buffered formalin for 10 minutes. Cells were incubated with the same primary antibodies described above for Wnt5a, Ror-2 and CTHRC-1 for one hour at room temperature. The secondary antibody used for Wnt5a and CTHRC1 was goat polyclonal anti mouse IgG Alexa Flour 568 and for Ror2 was goat polyclonal anti rabbit IgG Alexa Flour 488, incubated for 30 minutes. Slides were washed, stained with DAPI and visualized on a Nikon Eclipse A1 Ti-E confocal microscope. For better visualization of Wnt5a expression in the three cell lines, the 568 emission spectra was changed to 488 using the NIS Elements Ar Imaging software (Nikon).

### Statistical analyses

Logistic regression statistical analysis was performed to investigate the correlation between Wnt5a immunostaining and Ror2 immunostaining, CTHRC1 immunostaining, tumor histological grade, and pathological stage. Statistical significance was tested at an alpha of 0.05. The software PASW Statistics 18 was used for data analysis (Pearson Education, New York City, NY). Real-time RT PCR results are expressed as mean ± SEM. They were analyzed statistically using one-way analysis of variance (ANOVA) comparing RT4, J82 and T24 normalized expression values followed by Tukey post-hoc analyses. Results were considered statistically significant at *P* < 0.05.
